# How novel is protactinium: Insights into the structure and properties of (PaO)_2_(SO_4_)_3_(H_2_O)_2_

**DOI:** 10.1126/sciadv.adt7782

**Published:** 2025-04-30

**Authors:** Jarrod M. Gogolski, Garret Gotthelf, Megan E. Hoover, Vinh T. Nguyen, Binod K. Rai, Lindsay E. Roy, Thomas C. Shehee

**Affiliations:** Savannah River National Laboratory, Aiken, SC 29808, USA.

## Abstract

Because of the scarcity of protactinium and the challenges associated with its separation and crystallization, even in sulfuric acid media where protactinium is relatively stable, there has been an incomplete understanding of the structural features of protactinium complexes. The characterization of protactinium sulfate complexes has been limited to those in solution, which have left key details unaddressed since the 1960s. This report describes a synthetic strategy to crystallize a protactinium complex using boric acid and sulfuric acid. Herein, the authors detail the results of hydrothermal synthesis and the single-crystal analysis of a novel protactinium sulfate complex, (PaO)_2_(SO_4_)_3_(H_2_O)_2_. This work has elucidated structural features, providing groundwork for accurate computational analysis and clarifying previously unknown details on the coordination, denticity, and binding properties of protactinium sulfate complexes.

## Teaser

A rare and novel protactinium single crystal is synthesized, isolated, and characterized.

## INTRODUCTION

Protactinium (Pa) remains one of the rarest and least explored elements. Despite being a naturally occurring element, Pa was not identified until 1913. Fajans and Göhring (*1*) initially discovered an element, coined as “brevium” because of its brief half-life, which is now known as an isotope of Pa, Pa-234m. It was not until 1918 when Meitner and Hahn (*2*) discovered an isotope with a longer half-life, Pa-231. Although Pa is commonly found in uranium ores and as an intermediate in irradiated thorium fuels (*3*), research on Pa has been hindered by several challenges. Foremost, the global supply of Pa is scarce, requiring a substantial effort to isolate small quantities. The recovery of considerable quantities of protactinium, like the 100-g recovery in 1962, has allowed for limited research (*4*); however, this supply has dwindled to scant legacy sources.

Second, as noted by Fajans and Göhring, many Pa isotopes have half-lives shorter than a month. Separation and characterization techniques with longer experimental timelines, such as recrystallizations, become less efficient with Pa complexes. Only Pa-231 has a reasonable half-life for extended research. However, the daughter products of Pa-231 have high-energy gamma emissions that require specialized research facilities to minimize its negative health and environmental impact. Third, while Pa has proved invaluable for oceanography and geochronology studies, there are fewer applications (*5*) when compared to other actinides, such as U or Pu. However, this can be attributed to Pa’s scarcity and limited research on its properties.

Pa is distinguishable from the other actinides because of an inherently unique property. The location of Pa within the actinide series lies at a junction where the 6d and 5f orbitals cross in energy because of relativistic effects (*6*). Lighter actinides often exhibit transition metal–like behaviors that differ from those of heavier actinides (*7*). The near degeneracy between the 6d and 5f orbitals results in Pa complexes that can exhibit properties akin to both transition metal and actinide complexes. This duality is exclusive to Pa and presents a rich landscape for future exploration. Although Wilson (*8*) recently noted this particular Pa chemistry, few research institutions have the resources to further explore this unique behavior. Here, at Savannah River National Laboratory (SRNL), our research team has discovered a source of Pa-231 in a sealed container of uncertain origin. Isolation, analytical characterization, and computational analysis of a novel Pa sulfate complex are discussed herein.

## RESULTS AND DISCUSSION

### Isolation of Pa from a sealed source

**Fig. 1.**
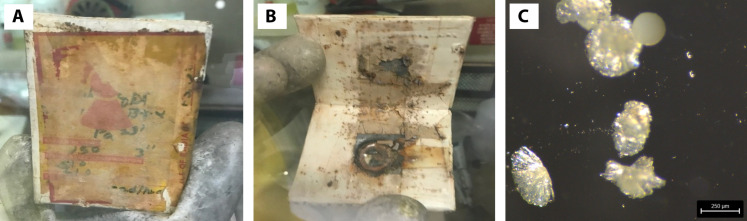
Legacy material containing Pa-231. (**A**) Lead storage container labeled with Pa-231. (**B**) Lead sheets containing the sample mixture. (**C**) Solids from the hydrothermolysis of Pa-231 and boric acid in a sulfuric acid solution.

A legacy material at SRNL was found stored in a lead container (Fig. 1A) and determined via gamma spectroscopy to contain Pa-231. The aging storage container and condition of the legacy material suggested that Pa-231 was contaminated by lead carbonate, metal impurities from a metallic washer, and organic impurities from a resin material (Fig. 1B). To collect all the available Pa-231 from the source, parts of a procedure published in (*9*) were done for the initial separation. Dissolving the legacy sample in hydrofluoric acid, collecting the liquid portion, and neutralizing with ammonium hydroxide allowed for the precipitation of solids containing Pa.

**Fig. 2.**
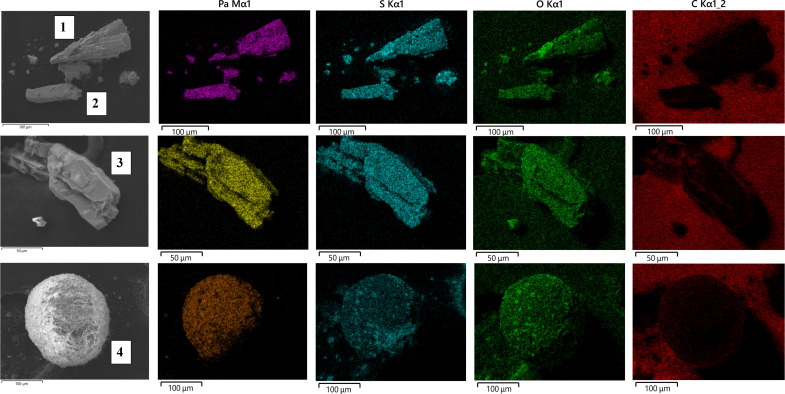
FIB-SEM images of samples 1 to 4 with EDS analysis. Selected elements (Pa, S, O, and C) were visualized.

Although underused, crystallization can be an effective pathway to isolate Pa complexes (*10*, *11*). However, the chemical nature of Pa presents challenges for recrystallization. Pa complexes are relatively unstable in solution and will often hydrolyze and adhere to reaction vessel surfaces, making recovery difficult (*3*). Fortunately, evidence suggests that sulfuric acid solutions help mitigate hydrolysis (*12*, *13*). The authors aimed to crystallize and characterize a Pa(V) borate crystal using a solution of sulfuric acid. Although Pa does not form a known hexavalent species, the experimental strategy described herein sought to replicate other work with thorium, uranium, neptunium, and plutonium (*14*, *15*) to form an analogous compound for comparison. To this end, the Pa precipitates and boric acid were dissolved in sulfuric acid. This reaction mixture was thermolyzed in a pressure vessel at 200°C for 1 week. Slow cooling of the resulting reaction mixture yielded two types of visually distinct solids. Several crystalline solids (samples 1 to 3) and one of the spherical clusters (sample 4) were collected without rinsing (Figs. 1C and 2).

### FIB-SEM and powder XRD analysis

Samples 1 to 4 were imaged by focused ion beam scanning electron microscopy (FIB-SEM), and their elemental compositions were quantified by energy-dispersive x-ray spectroscopy (EDS) (Fig. 2, figs. S2 to S6, and table S1). The crystalline solids, samples 1 to 3, were large single crystals on the scale of 50 by 100 μm that were suitable for single-crystal x-ray diffraction (XRD). A detailed image of the spherical cluster, sample 4, revealed that the sphere was not a single entity but rather an amalgamation of crystals less than 4 by 5 μm in size. A powder XRD analysis of sample 4 revealed diffraction patterns matching iron sulfates and metaborite but did not contain patterns matching known Pa structures [i.e., H_3_Pa(SO_4_)_3_] that could have formed in this solution (*16*).

Elemental analysis by EDS indicated that Pa, oxygen, and sulfur were well distributed throughout all four samples. The calculated S:Pa ratio from the elemental compositions indicated that samples 1 to 3 had S:Pa ratios between 1.5 and 1.7, while the ratio for sample 4 was 1.0 (see table S1). These ratios suggested sulfate coordination to Pa and that samples 1 to 3 could be the same complex. Although oxygen was present throughout samples 1 to 4, the elemental composition of the FIB-SEM sample holder, primarily oxygen and carbon (table S2), prevented an accurate quantitation of oxygen in the samples. Negligible amounts of carbon were detected on the solids.

Trace amounts of boron could be faintly visualized in the background of the FIB-SEM images but not on the solids themselves. In contrast, nitrogen was unequally distributed throughout samples 1 to 4 but not detected in the background. Although a Pa borate complex was targeted, the absence of boron indicated that borate was not a constituent of samples 1 to 4. Given boric acid can be consumed by residual hydrofluoric acid or by thermolysis with sulfuric acid to form metaborite, the boric acid likely decomposed rather than complexing with Pa. Residual boron contamination in the sample background is likely due to not rinsing the solids before analysis.

For nitrogen, residual ammonium carried over from the preceding purification steps can explain its presence on the solids. However, powder XRD data collected from sample 4 did not contain patterns matching that of any known nitrogen-containing compounds (fig. S7). Likely, an unidentified Pa-ammonium compound formed. However, given the unequal distribution of nitrogen on the single-crystal samples, the Pa-ammonium compound could be sporadically present as a passivating surface layer rather than as a constituent of the crystal unit cell. In this scenario, the measured nitrogen composition would be overrepresented because EDS detection skews toward elemental analysis of the surface layers of the solids.

Trace amounts of iron, aluminum, and silicon were detected in some samples. Presumably, these elements were contaminants that originated from the dissolution of the legacy sample and the metallic washer. Powder XRD patterns confirmed the presence of iron sulfate hydrates (fig. S7) that would account for iron contamination. Oxides, such as Al_2_O_3_ or SiO_2_, were likely contaminants but were too low in concentrations to be distinguishable via powder XRD.

### Single-crystal XRD analysis

**Fig. 3.**
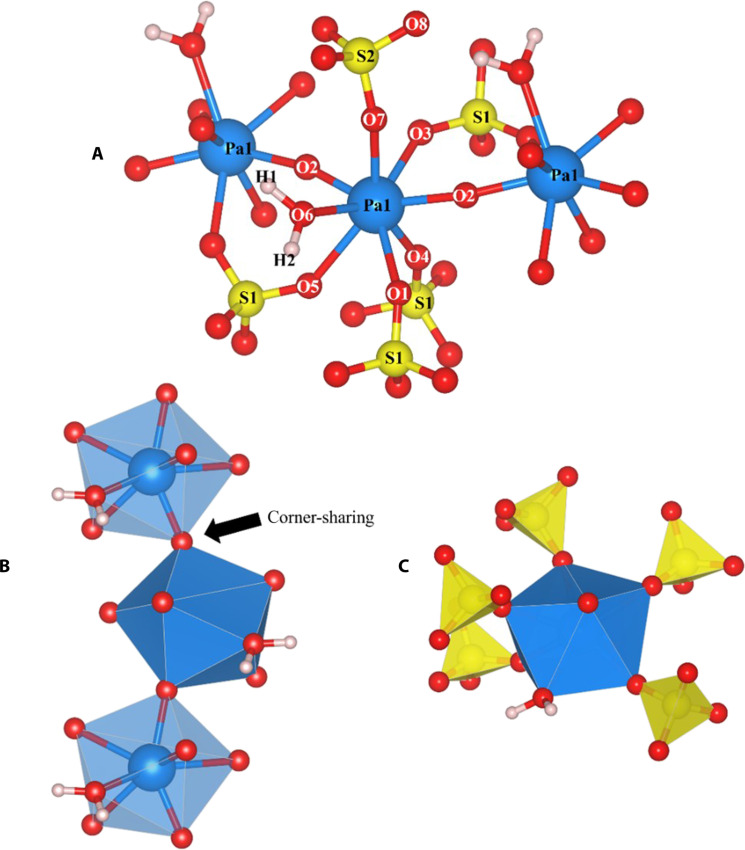
Crystal structure of Pa_2_S_3_H_4_O_16_. (**A**) The local coordination around the Pa-atom center was highlighted, and the atom labels correlated to those in Table 1. Polyhedral depiction of (**B**) the bridging Pa structure and (**C**) the coordination around the Pa-atom center. The atom colors correspond to the following: Pa, blue; S, yellow; O, red; H, white.

Single-crystal XRD analysis of a crystalline sample revealed a monoclinic cell with a *C*2/*c* space group, a density of 4.6640 g/cm^3^, and an empirical formula of Pa_2_S_3_H_4_O_16_. Lattice constants for the unit cell were determined to be the following: *a* = 22.2345(4) Å, *b* = 6.6587(1) Å, *c* = 7.9279(1) Å, α = γ = 90°, and β = 96.894(2)°. The eight-coordinate Pa centers adopted a distorted bicapped trigonal prismatic geometry where the ligand vertices were arranged by two distorted biaugmented triangular prisms with 10 triangle faces and one square face (Fig. 3). The empirical formula indicated a Pa(V) center, which has been noted to be the most stable state for Pa (*8*).

**Fig. 4.**
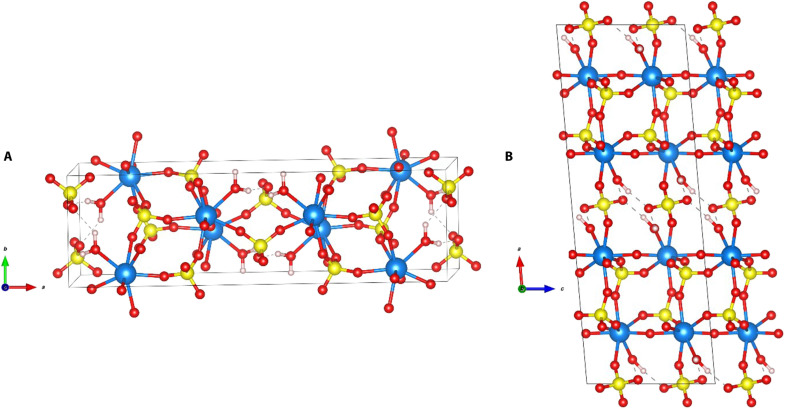
Multiple visual representations of the distorted bicapped trigonal prismatic geometry of the eight-coordinated Pa were depicted for readability. Using density functional theory, the XRD crystal structure was optimized to allow the atoms and cell volume to completely relax to determine their lowest energy geometry. The atom colors are as follows: Pa, blue; S, yellow; O, red; H, white. Perspective viewed (**A**) along the *c* axis within the *ab*-plane and (**B**) along the *b* axis within the *ac*-plane of the (PaO)_2_(SO_4_)_3_(H_2_O)_2_ unit cell.

When viewed along the *b* axis (Fig. 4B), Pa–O bonds can be seen bridging Pa centers together into a chain-like pattern, with an O–Pa–O angle of 147.5°. As seen by the bridging oxygens, the isolated structure is polymeric with the corner-sharing oxygens between the bipyramids having a Pa–O bond of 2.004(3) Å and the oxygens on the square face having a Pa–O bond of 2.143(3) Å (Fig. 3B). Bond lengths for the sulfate Pa–O [2.257(4) to 2.451(4) Å] bonds and the bridging Pa–O bonds were well below the sum of the Pa–O radii, which both agree well with our theoretical calculations for the optimized structure (Table 1). Although most single-crystal XRD characterizations have been limited to the Pa metal, oxides, and halides (*11*, *17*), an estimated covalent Pa–O radius of 2.66 Å indicated that all Pa–O bonds were well within the expected bond distance (*18*, *19*).

**Table 1.** Experimental and calculated bond distances between selected atoms. DFT, density functional theory.

Atom 1	Atom 2	Distance (Å)	DFT distance (Å)
Pa1	O1	2.388(3)	2.384
	O2	2.004(3)	2.080
		2.143(3)	2.138
	O3	2.408(3)	2.402
	O4	2.287(3)	2.310
	O5	2.416(3)	2.423
	O6	2.451(4)	2.436
	O7	2.257(4)	2.278
S2	O7	1.492(4)	1.494
	O8	1.443(4)	1.467
O6	O7	2.699(5)	2.723
	O8	2.764(5)	2.664

Pentavalent actinide oxo complexes, such as those containing uranium, neptunium, plutonium, and americium, generally form linear dioxo complexes (*20*, *21*). Characterized as having multiple bond orders, these An–O bonds can bridge with other actinide centers through nucleophilic oxygens. The bridging oxygens often coordinate at the equatorial vertices of other actinide centers via a coordination type coined as cation-cation interactions (*21*–*23*). Despite the observed conformation of the dioxo complex for other pentavalent actinides, a Pa dioxo species has yet to be isolated. Some sources calculate a stable gas phase PaO_2_^+^ (*24*), but many studies suggest that aqueous Pa species prefer alternative conformations, such as a mono-oxo structure (*25*, *26*). To date, mono-oxo Pa complexes have been observed in aqueous solutions, with only one example of a crystal (*17*, *21*). Bridging Pa–O structures have also been reported and include those with oxygen bridging three or more Pa centers, such as Wilson’s recent peroxo cluster (*6*, *27*). However, these structures do not involve sulfate ligands and are often stabilized by halogens.

For the isolated (PaO)_2_(SO_4_)_3_(H_2_O)_2_ complex in this study, the Pa–O–Pa oxygen, denoted as O2 in Fig. 3A, can be classified as a bridging oxygen. The geometry of the Pa complex differs from the typical bipyramidal geometry of linear dioxo complexes, and the resulting O2–Pa–O2 angle (147.5°) significantly deviates from linearity. The bridging oxygen Pa–O bond has a length of 2.004(3)/2.143(3) Å, which is longer than the bond lengths of a multiple bonded Pa–O mono-oxo or dioxo bond (*28*–*30*).

In previous studies of mono-oxo Pa sulfate species, Le Naour *et al.* (*31*) and Mendes *et al.* (*32*) determined Pa–O bond lengths of 1.72 and 1.75 Å, respectively. These values match well with Toraishi’s calculations indicating mono-oxo Pa bond lengths between 1.718 and 1.799 Å (*26*), although recent studies have suggested that the calculated value may be longer than previously thought (*33*). These mono-oxo Pa–O bonds, which have been characterized as akin to triple bonds (*34*, *35*), are significantly shorter than the Pa–O2 bonds [2.004(3)/2.143(3) Å] in this study. Calculations by Dixon and co-workers (*36*) predicated Pa–O bond lengths for various hydrolysis intermediates of Pa complexes to be within the range of 2.1 to 2.4 Å for Pa-hydroxy and bridging Pa–O–Pa complexes (*36*). Furthermore, a PaO_2_(OH) structure was calculated to have a Pa–OH bond length of 2.159 Å. The Pa–O2 bond length for (PaO)_2_(SO_4_)_3_(H_2_O)_2_ falls within or below the range for these calculated intermediates.

To understand the bonding characteristics of this material from a computational chemistry standpoint, the crystal orbital bond index (COBI) is used to measure the strength of covalency on the bond (*37*). This technique, which typically describes the chemical bond strength in molecules, has been extended to include crystalline materials and is based on the bond index by Wiberg and Mayer. As the integrated COBI (ICOBI) values decrease, ionicity increases. In this work, the ICOBI values for the eight Pa–O distances and six unique S–O distances are listed in Table 2.

**Table 2.** Calculated bond distances between selected atoms and ICOBI values.

Atom 1	Atom 2	DFT distance (Å)	ICOBI value
Pa1	O1	2.384	0.23
	O2	2.080	0.61
		2.138	0.51
	O3	2.402	0.22
	O4	2.310	0.28
	O5	2.423	0.20
	O6	2.436	0.22
	O7	2.278	0.30
S2	O4	1.491	0.46
	O7	1.494	0.44
	O8	1.467	0.53
	O9	1.479	0.48
	O10	1.476	0.49
	O11	1.469	0.50

The Löwdin charges for Pa^5+^, S^6+^, O^2−^, and H^+^ are +1.58 *e*, +2.52/+2.57 *e*, −0.50 to −0.88 *e*, and +0.40 *e*, respectively. The S–O bonds where the oxygens also participate in hydrogen bonding had a decreased Löwdin charge of +2.52 *e* compared to the other sulfate ion. The Löwdin charges were −0.50 *e* and −0.81 *e* to −0.88 *e* for the oxygen atoms within the Pa–O mono-oxo chain and oxygen atoms bonded to sulfur, respectively. For the waters, the oxygen Löwdin charge was −0.68 *e*. The ICOBI values for Pa–O_oxo_ are 0.51 and 0.61 for the Pa–O mono-oxo bond distances of 2.138 and 2.081 Å, respectively. The ICOBI values indicated primarily covalent contributions. These exhibit a weaker contribution of covalency for Pa–O bonds where the oxygen atoms also bond to the sulfate ions or water (0.20 to 0.30). Last, the ICOBI values are 0.44 to 0.50 and 0.53 for the S–O bonds where the O is also bonded with the Pa atom and where the oxygen is participating in hydrogen bonding, respectively. These ICOBI values do hint at there being both ionic and covalent character to the S–O bonds in the sulfate ions.

Apart from Pa–O2, there are five sulfate anions coordinated to each Pa(V) center in a monodentate manner (Fig. 3C). These five sulfate anions can be divided into two inequivalent types of sulfate anions. The first type of sulfate anion (labeled S1 in Fig. 3A), which constitutes four of the five coordinated sulfates, binds to four different Pa(V) centers. The second type of sulfate anion (labeled S2 in Fig. 3A), which constitutes one of the five coordinated sulfates, only coordinates to two Pa(V) centers. This second type of sulfate anion has two seemingly uncoordinated S–O bonds oriented toward a void in the crystal structure. Water molecules were found to reside in this void and were bound to the Pa(V) center in a 1:1 Pa:H_2_O ratio. Computations optimizing this structure determined that one of the water O–H bonds was parallel with the *b* axis plane, creating a larger void (Fig. 4A).

Previous examples of Pa sulfate species have been hypothesized to be hexacoordinated or involve bidentate sulfates (*38*, *39*). In contrast, our structure had an eight-coordinate Pa(V) center and monodentate sulfate anions for (PaO)_2_(SO_4_)_3_(H_2_O)_2_. The larger coordination number of the Pa center may contribute to a greater degree of steric crowding and elongated Pa–O bond distances than previously hypothesized. However, it should be noted that measurements of lattice bond distances can significantly differ from those determined from complexes in solution (*40*). Since prior structural characterizations of Pa sulfate complexes have been limited to computations, PXRD, or samples in solution (extended x-ray absorption fine structure and x-ray absorption near-edge structure), only partial structural elucidations have been possible (*11*, *16*, *31*, *38*, *39*, *41*). Previous studies on sulfate and water coordination to Pa(V) centers have offered valuable insight; however, the single-crystal characterization of (PaO)_2_(SO_4_)_3_(H_2_O)_2_ offers deeper structural insight.

As alluded to by some authors, a lack of experimental characterizations of Pa(V) complexes has resulted in greater reliance on hypothesized behaviors to identify structural features (*39*). Ambiguity regarding the extent of water coordination and the denticity and number of sulfate ligands has been noted (*25*, *31*). Single-crystal XRD analysis of (PaO)_2_(SO_4_)_3_(H_2_O)_2_ and the supporting computational analysis of this structure provided a Pa complex with an unambiguous ligand environment. As reported in Table 1, the Pa–O bonds to the S1 sulfate oxygens (O1 and O3 to O5) have bond distances between 2.257(4) and 2.451(4) Å. These Pa–O bonds associated with the S1 sulfate are longer than the Pa1–O7 bond, 2.258(4) Å, to the S2 sulfate oxygen. A charge distribution among four Pa–O bonds, as opposed to two Pa–O bonds, resulted in a longer Pa–O bond for the S1 sulfate. The most elongated Pa–O bond, 2.451(4) Å, was between Pa and a bound water molecule and matched well with previous calculations for a Pa-bound water (*25*). Within the void, the distances between the O_water_ and the two neighboring O_sulfate_, or O6–O7 and O6–O8, were 2.699(5) and 2.764(5) Å, respectively. Given an expected O–H–O distance (2.7 Å) for sulfuric acid and water, H-bonding interactions were likely present (*42*).

This is a notable example of a single-crystal characterization of a Pa sulfate. To our knowledge, the most analogous Pa complex to (PaO)_2_(SO_4_)_3_(H_2_O)_2_ is (PaO)_2_(HPO_4_)_3_·2H_2_O and was precipitated by Le Cloarec (*43*). This precipitate was characterized by infrared spectroscopy and was believed to also contain a bridging Pa–O–Pa feature because of an infrared stretch at 610 cm^−1^. However, this complex was part of an amorphous solid and single-crystal XRD characterization was not performed.

### Density of states

**Fig. 5.**
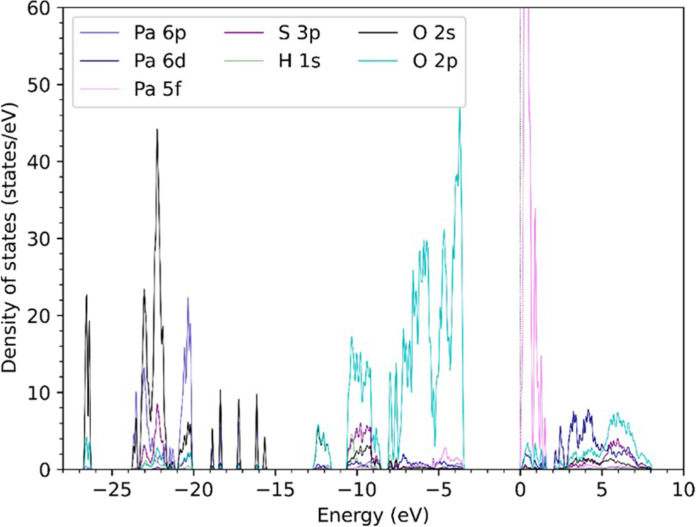
Density of states analysis. Electronic density of states for (PaO)_2_(SO_4_)_3_(H_2_O)_2_.

Calculation of the density of states confidently indicated a Pa(V) center (Fig. 5). A charge-transfer insulator with a bandgap of 3.5 eV separated the conduction and valence bands. Other protactinium oxides using the GGA + *U* method have predicted bandgaps of 3.48 eV (PaO_2_) (*44*), whereas the bandgaps reported by Siberchicot and Aupiais (*45*) were 2.85 eV for λ-Pa_2_O_5_ and 1.96 eV for ζ-Pa_2_O_5_. Hybridized O 2s and 2p atomic orbitals constituted the inner valence molecular orbitals and the outer valence molecular orbitals, respectively. The hybridization of the S 3p and O 2s/2p atomic orbitals occurred around −10.5 to −9.1 eV, while most of the Pa 6d and 5f atomic orbitals resided in the conduction band and supported a Pa(V) center. Given the stability of a Pa(V) state and the oxidizing conditions of a hydrothermal synthesis, the density of states data do not deviate from our expectations and supported the proposed empirical formula.

### Novelty of protactinium

The crystallization of this complex allowed for a novel opportunity for single-crystal XRD analysis of a Pa(V) sulfate species. Despite the initial strategy to synthesize a Pa borate complex, our serendipitous synthesis of (PaO)_2_(SO_4_)_3_(H_2_O)_2_ may indicate a fruitful path for future crystallizations. The experimental conditions presented herein may have been more ideal for product formation and crystallization. While the authors harbor reservations on the reasoning, the degradation of boric acid in a sulfuric acid solution, and the concurrent increase in acidity, may create more optimal recrystallization conditions. However, a combination of acidity and sulfate anions may allow for stable complexations of Pa, especially given that (PaO)_2_(SO_4_)_3_(H_2_O)_2_ was unique even among other observed Pa(V) compounds obtained from acidic media. While previous Pa(V) compounds were observed to have their charge balanced by protons (*16*), the synthesized Pa(V) crystal was instead balanced by an additional bridging Pa(V)–O bond. Although the species, (PaO)_2_(SO_4_)_3_(H_2_O)_2_, is without much doubt one of many Pa sulfate complexes, this example confirmed key structural features that have been the topic of debate since the 1960s.

## MATERIALS AND METHODS

### Radiological precautions

Caution, because of the high radiotoxicity, all work with Pa-231 was done in negative-pressure glove boxes, fume hoods, or environments approved for handling high-activity radiological materials.

### Reagents and equipment

Boric acid and hydrofluoric acid were purchased from Spectrum Chemical Manufacturing Corporation. Concentrated sulfuric acid and ammonium hydroxide were purchased from Thermo Fisher Scientific. A 23-ml acid digestion vessel (Parr vessel) with a Teflon liner was purchased from the Parr Instrument Company.

### Synthesis of (PaO)_2_(SO_4_)_3_(H_2_O)_2_ from legacy materials

A legacy material at SRNL was found stored in a lead container and was assessed via gamma spectroscopy to contain Pa-231 (Fig. 1). Inside the container, three sheets of lead were individually wrapped in vinyl tape and then further taped together to form a container. Removing some tape and lifting the top sheet of lead revealed a well containing a mixture of an unidentified sample, decomposed tape, a relatively untarnished metal washer, and a white powder believed to be lead carbonate. The washer and sample had a sheen, suggesting that the material may have been coated.

To isolate a sample of Pa-231, the washer and sample were first stirred in 10 ml of 5 M hydrofluoric acid solution for 10 min. The liquid portion was decanted and then neutralized by a dropwise addition of 3.5 ml of a 14.5 M NH_4_OH solution. After centrifuging the reaction mixture, the brown supernatant was decanted, and the white precipitates were collected. The precipitates were redissolved in 5 ml of a 3 M sulfuric acid solution to form a stock solution. An aliquot was taken for gamma spectroscopic analysis using an HPGe coaxial gamma detector and counted for 4 hours, which indicated that Pa-231 (1.83 × 10^8^ dpm/ml) and its daughter product Th-227 (1.37 × 10^7^ dpm/ml) were recovered. The intermediate radionuclide Ac-227 is undoubtedly present; however, its gamma energies are quite low and have some overlap with Th-227. Trace quantities of other radionuclides (e.g., 2.39 × 10^4^ dpm/ml of Cs-137) seemed to be present with the sample solution, but their origin is uncertain. For instance, Cs-137 would be expected from fission reactions, which could have occurred if the Pa-231 was prepared by neutron irradiation of Th-230 (*46*). The working stock contained Pa-231 (1.83 mg/ml) based on activity listed above.

A Parr vessel was charged with 14.7 mg of boric acid and 1 ml of the Pa-231 sample solution. The Parr vessel was sealed, heated at 200°C for 7 days inside a muffle furnace, and then allowed to cool to room temperature. Several crystals and spherical solids were collected without rinsing. Crystals for single-crystal XRD and a spherical cluster for powder XRD were collected for analysis. Representative samples were also selected for analysis by FIB-SEM.

### Electronic structure calculations

Quantum-mechanical calculations were executed using density functional theory (*47*, *48*) with the projector augmented-wave method (*49*, *50*), as implemented in the Vienna Ab initio Simulation Package (*51*–*54*). The generalized gradient approximation with the modified Perdew-Burke-Ernzerhof exchange-correlation functional for solids (*55*) is used for all calculations. The XRD data provided by the experimentalists in this work are used as the initial geometry for the relaxation calculations. To capture the on-site Coulomb repulsion between the 5f electrons, the rotationally invariant Hubbard *U* correction term (GGA + *U*) is applied to the protactinium f electrons using the Liechtenstein method (*56*), where *U* = 4.0 eV and *J* = 0.0 eV. A plane-wave cutoff energy of 520 eV is applied to all calculations with an electronic convergence and force convergence of 10^−6^ and 10^−3^, respectively. The Monkhorst-Pack scheme is used for the *k*-point sampling of the Brillouin zone with the smallest allowed *k*-point spacing equaled to 0.3 Å^−1^. Gaussian smearing is used with a smearing width of 0.05 eV.

### Single-crystal x-ray diffraction

X-ray crystallography data were collected using a Rigaku XtalLAB Synergy diffractometer. This equipment features a HyPix 3000-pixel array detector and a microfocus sealed tube (Mo-Kα radiation at a wavelength of 0.71073 Å). It operated under the conditions of 50 kV and 1 mA. The measurements were taken at room temperature (299.5 K) using colorless single crystals. These crystals were mounted on a Mitigen Cryoloop. To ensure the data’s completeness and achieve the desired level of redundancy, CrysAlisPro software was used (*57*). This program guided the strategy for data collection, the actual data collection process, and the subsequent data processing. An empirical absorption correction was applied to the collected data using spherical harmonics, as implemented in the SCALE3 ABSPACK scaling algorithm. The initial solution to the crystal structure was found using the ShelXT structure solution program using a dual solution method with the Olex2 graphical interface (*58*, *59*). Final refinement of the model was done with ShelXL (*60*). This refinement used a full-matrix least-squares method based on *F*^2^ minimization.

### Powder x-ray diffraction

Powder x-ray diffraction patterns were collected using a Rigaku XtalLAB Synergy diffractometer. This equipment features a HyPix 3000-pixel array detector and a microfocus sealed tube (Mo-Kα radiation at a wavelength of 0.71073 Å). It operated under the conditions of 50 kV and 1 mA. A microgram sample of the spherical cluster, sample 4, was mounted in a MiTeGen loop, and measurements were taken at room temperature (299.5 K). To ensure the homogeneity of samples, a Gandolfi move for powders and data were collected from 2θ values from 3° to 52°.

### Focused ion beam scanning electron microscope

A Zeiss Crossbeam 550 focused ion beam scanning electron microscope can be used as a traditional scanning electron microscope, with accelerating voltages from 20 to 30,000 V and currents from 10 pA to 40 nA. The secondary electron secondary ion detector was used for imaging. The focused ion beam scanning electron microscope included an Oxford Instruments Ultim 100 EDS detector for detection of x-rays from elements. Oxford Instruments Aztec 4.4 data acquisition and analysis suite was used to collect EDS data and complementary images, including a mapping capability.
