# Supplementary material for: How novel is protactinium: Insights into the structure and properties of (PaO)2(SO4)3(H2O)2
Source: Sci Adv. 2025 Apr 30;11(18):eadt7782. doi: 10.1126/sciadv.adt7782 (PMC12042872; doi:10.1126/sciadv.adt7782)
Supplement: Supplementary file 1 — Figs. S1 to S7 Tables S1 and S2 [file sciadv.adt7782_sm.pdf]

Science Advances
AAAS

Supplementary Materials for

How novel is protactinium: Insights into the structure and properties of $(PaO)_2(SO_4)_3(H_2O)_2$

Jarrod M. Gogolski *et al.*

Corresponding author: Jarrod M. Gogolski, jarrod.gogolski@srnl.doe.gov

Sci. Adv. **11**, eadt7782 (2025)
DOI: 10.1126/sciadv.adt7782

This PDF file includes:

Figs. S1 to S7
Tables S1 and S2

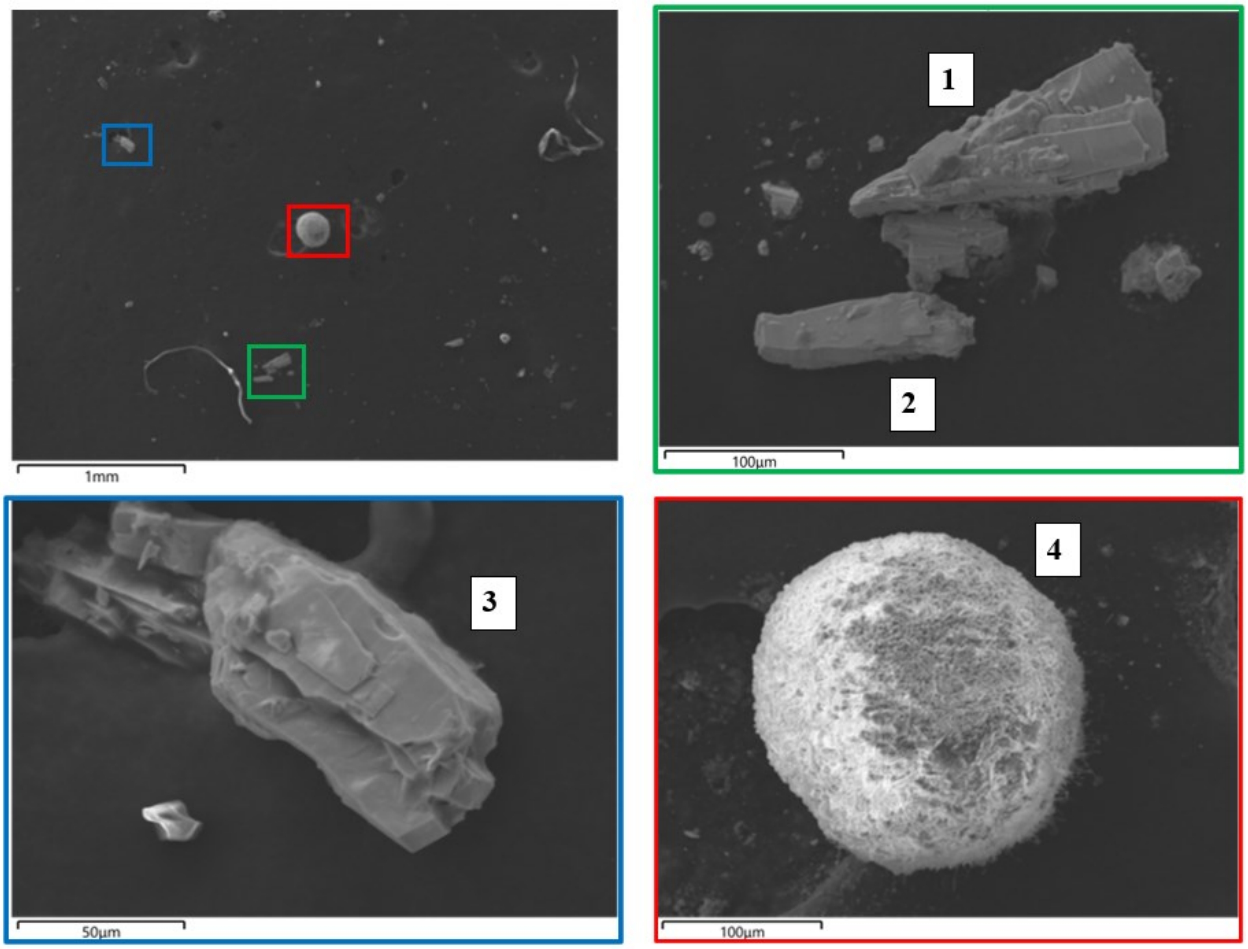

Fig. S1. FIB-SEM images of isolated crystals (sample 1-3) and a spherical cluster (sample 4).

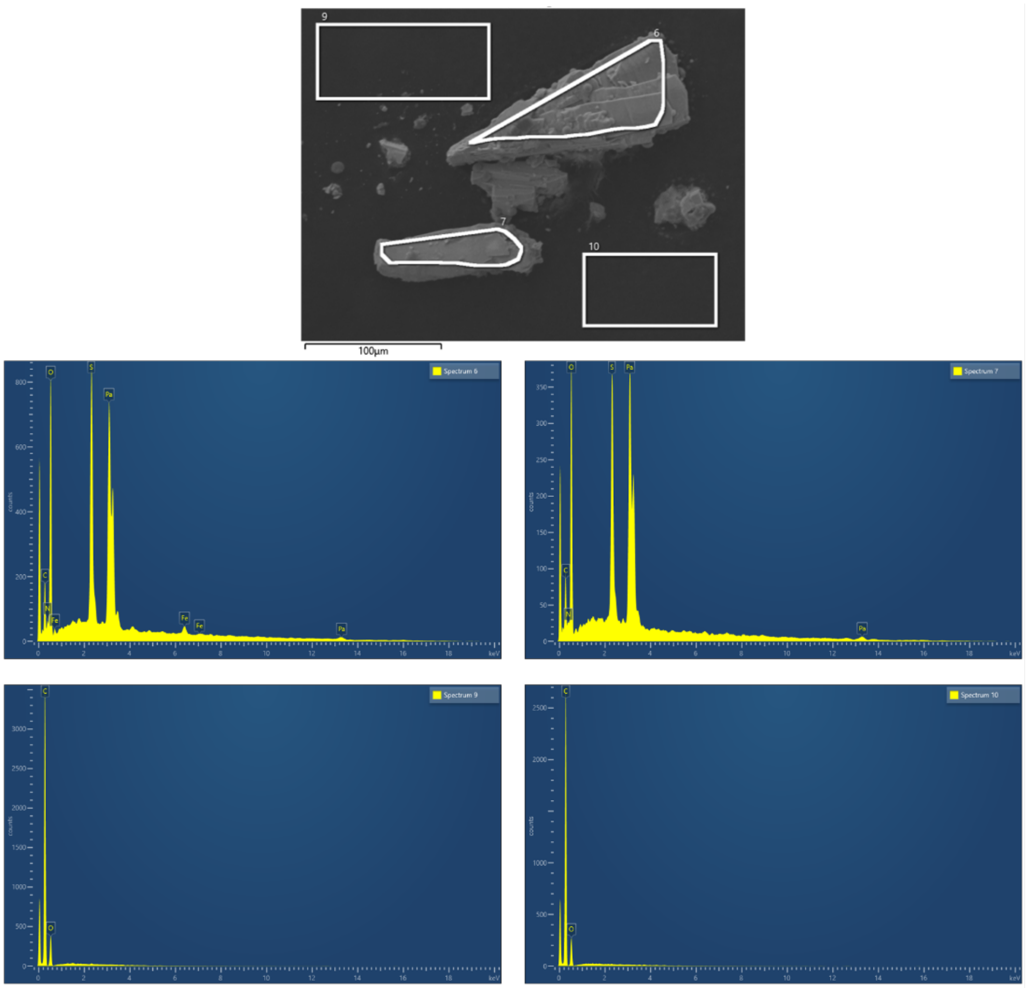

Fig. S2. FIB-SEM image of crystals (sample 1 and 2) with EDS data for highlighted regions 6, 7, 9, and 10.

Table S1. Relative elemental percent compositions and elemental ratios of the selected regions for samples 1-4.

Sample	Pa	S	O	N	Al	Si	Fe	S:Pa	O:Pa
1	7.2	12.5	73.0	6.4	-	-	1.0	1.7	10.2
2	8.0	11.8	76.1	4.1	-	-	-	1.5	9.5
3	6.8	10.7	73.8	7.9	0.1	0.6	-	1.6	10.8
4	9.1	9.5	71.0	8.1	0.4	1.2	0.7	1.0	7.8

Table S2. Relative elemental percent compositions of highlighted regions 9 and 10.

Region	C	O
9	81.0	19.0
10	81.5	18.5

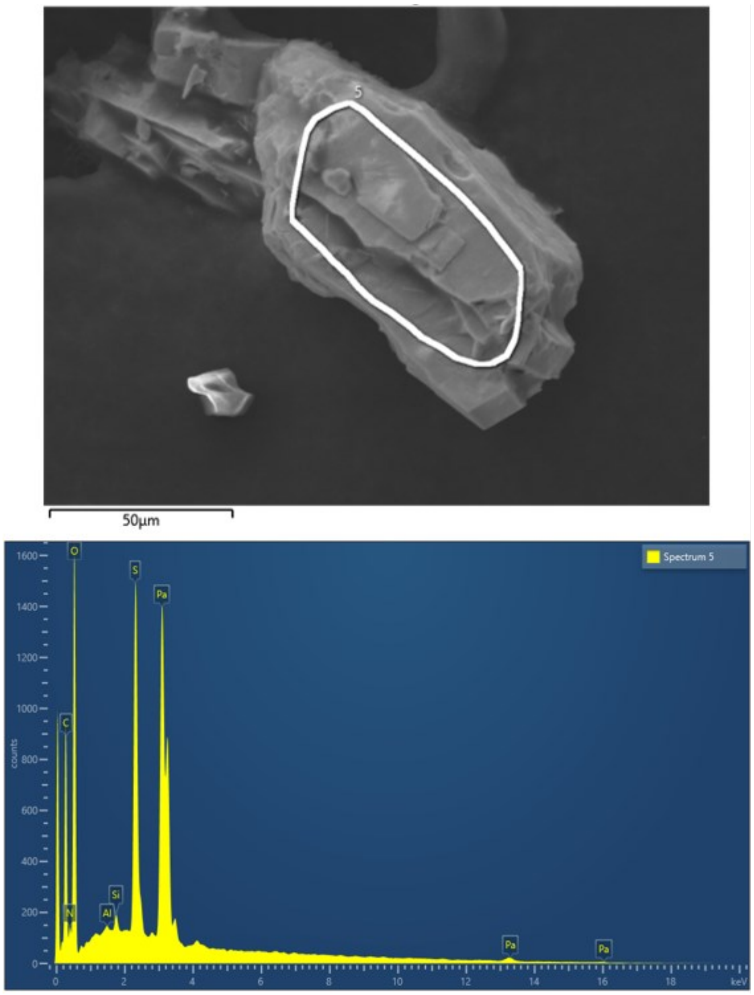

Fig. S3. FIB-SEM image of a crystal (sample 3) with EDS data for highlighted region 5.

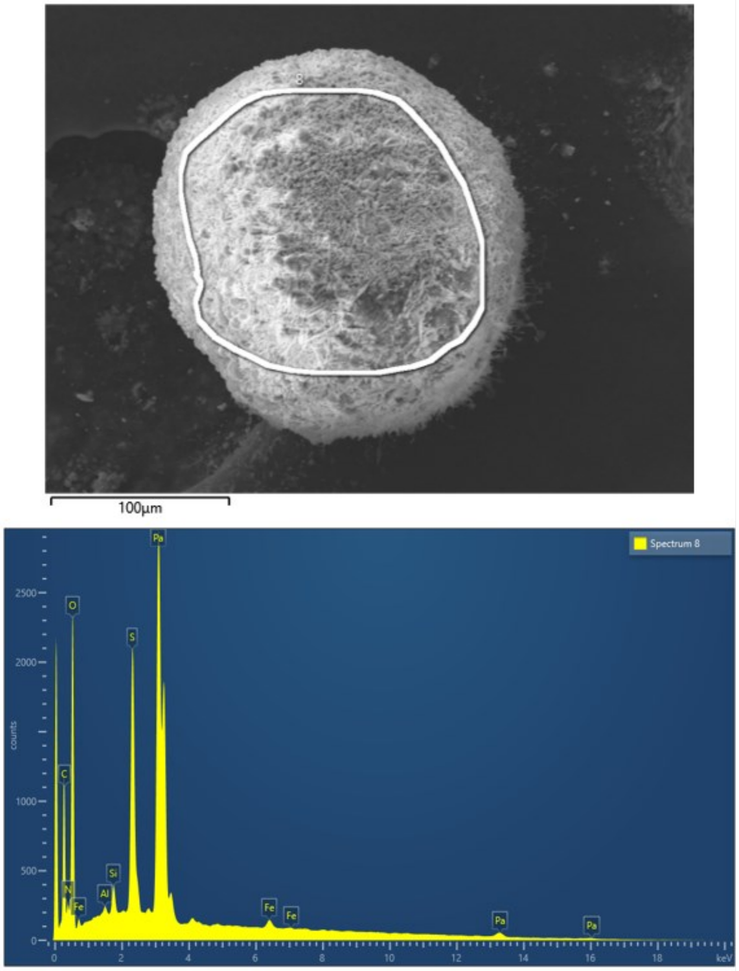

Fig. S4. FIB-SEM image of a spherical solid (sample 4) with EDS data for highlighted region 8.

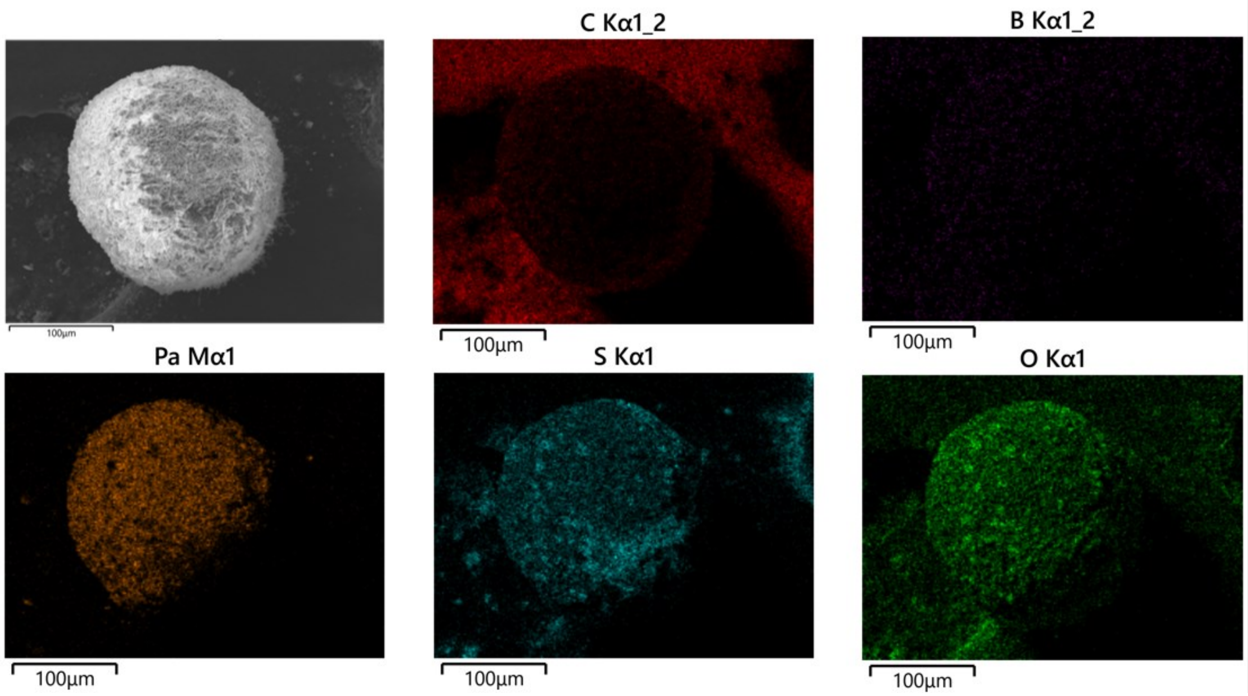

Fig. S5. FIB-SEM images of the spherical cluster, sample 4, visualizing C, B, Pa, S, and O.

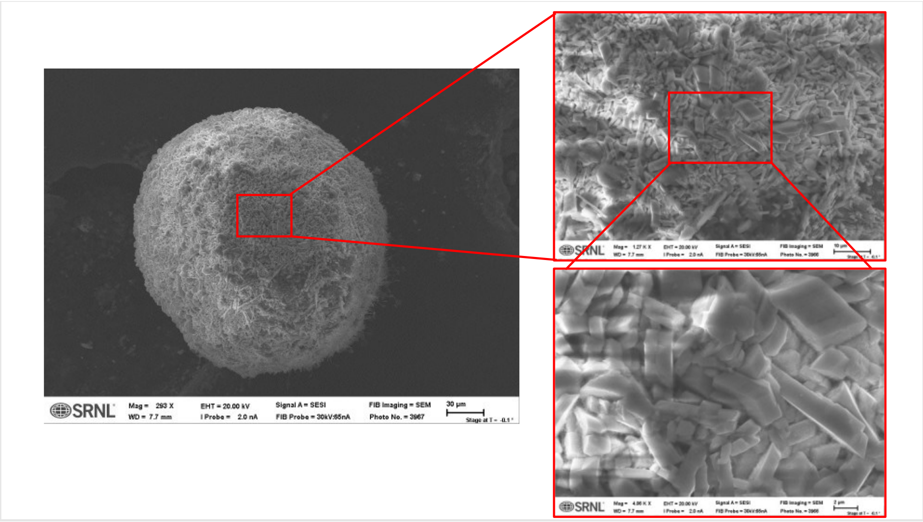

Fig. S6. Zoomed view of the FIB-SEM image of sample 4.

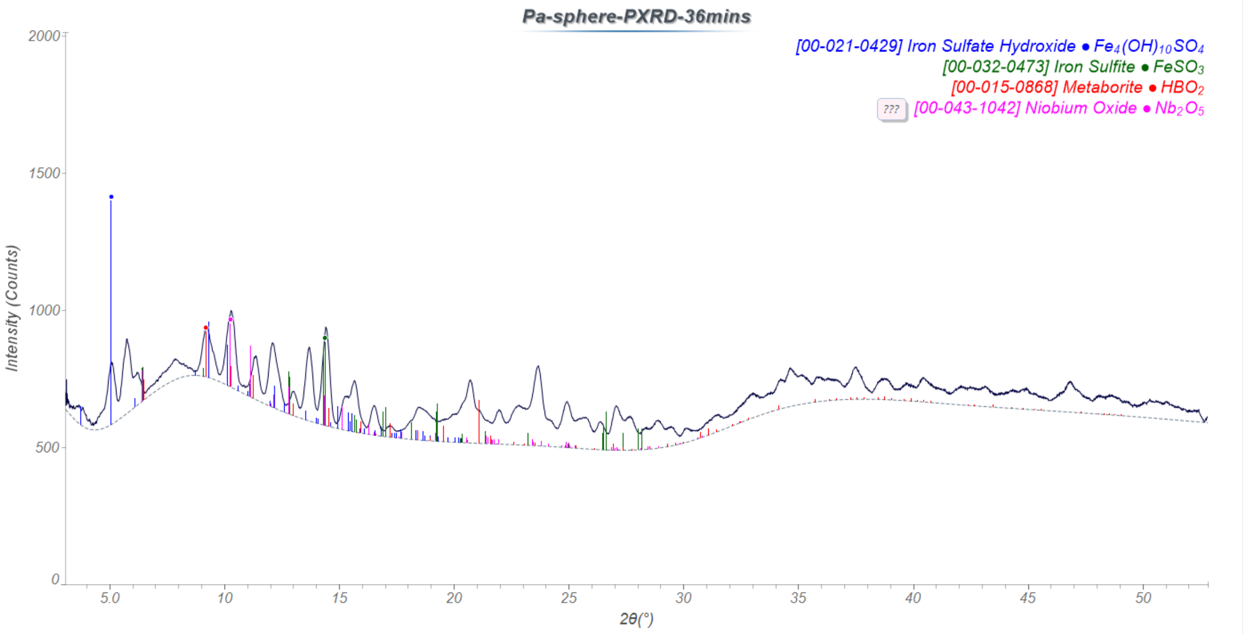

Fig. S7. Powder XRD data of the spherical cluster (sample 4) overlayed with the powder XRD data of several potential impurities.
